# Silliman’s Journal

**Published:** 1851-01

**Authors:** 


Art. VIII.—*Silliman's Journal*—Published at New Haven, Connecticut, every two months in numbers of 152 pages, at $5 a year in advance.

Contents of number thirty-one, for January, 1871:

"Art. 1.—Comparison of Experiments on American and Foreign Building Stones to determine their relative strength and durability; by Prof. Walter R. Johnson.

"Art. 2.—On the Law of the Induction of an Electric Current upon itself when developed in a straight prismatic conductor, and of discharges of Machine Electricity through straight wires; by J. H. Lane.

"Art. 3.—On Meteorites; by Charles Upham Shepard.

"Art. 4.—An Essay on the Classification of Nemertes and Planariæ: preceded by some general considerations on the primary Divisions of the Animal Kingdom; by Charles Girard.

"Art. 5.—Memoir on Emery; by J. Lawrence Smith, M.D. Second Part—On the Minerals associated with Emery.

"Art. 6.—On the Velocity of the Galvanic Current in Telegraph Wires; by B. Gould, Jr., in a Report to Prof.

A. D. Bache, LL. D., Superintendent of the U. S. Coast Survey.

"Art. 7.—Reply to Mr. De la Rue's remarks on the Navicula Spencerii contained in the American Journal of Science, vol. ix, p. 23; with a notice of two new test-objects; by J. W. Bailey.

"Art. 8.—Miscellaneous Notices; by J. W. Bailey.

"Art 9.—On the time required to raise the Galvanic Current to its maximum in Coiled Conductors, and its importance in Electro-Mechanics; by Prof. Charles G. Page, M.D.

"Art. 10.—A new figure in Mica and other Phenomena of Polarized Light; by Professor Charles G. Page, M.D.

"Art. 11.—Description of new species of Fungi collected by the U. States Exploring Expedition under C. Wilkes, U. S. N., Commander; by Rev. M. J. Berkeley and Rev. M. A. Curtis.

"Art. 12.—On the markings of the Carapax of Crabs; by James D. Dana.

"Art. 13.—Chemical examination of a Phosphate of Iron, Manganese, and Lithia, from Norwich, Mass.; by W. J. Craw.

"Art. 14.—On the Physical and Crystallographic characters of the Phosphate of Iron, Manganese, and Lithia, of Norwich, Mass.; by James D. Dana.

"Art. 15.—Notice of the discovery by Walter Mantell, Esq., of Wellington, in the Middle Island of New Zealand, of a living specimen of Notornis, a bird of the Rail Family."

Besides, 50 pages are devoted to new discoveries in Chemistry, Physiology, Mineralogy, Astronomy, General Science, and Bibliographical Notices.

We cannot too warmly commend this work to our readers. It is an honor to science; a work of which every American may well be proud.

